# Preparation and Biological Evaluation of [^99m^Tc]Tc-CNGU as a PSMA-Targeted Radiotracer for the Imaging of Prostate Cancer

**DOI:** 10.3390/molecules25235548

**Published:** 2020-11-26

**Authors:** Di Xiao, Xiaojiang Duan, Qianqian Gan, Xuran Zhang, Junbo Zhang

**Affiliations:** 1 Key Laboratory of Radiopharmaceuticals of Ministry of Education, College of Chemistry, Beijing Normal University, Beijing 100875, China; 202031150054@mail.bnu.edu.cn (D.X.); 201731150054@mail.bnu.edu.cn (Q.G.); 201321150179@mail.bnu.edu.cn (X.Z.); 2 Department of Nuclear Medicine, Peking University First Hospital, Beijing 100034, China; duanxj@pku.edu.cn

**Keywords:** ^99m^Tc, PSMA, isonitrile, prostate cancer, SPECT imaging

## Abstract

Prostate-specific membrane antigen (PSMA) is a well-established biological target that is overexpressed on the surface of prostate cancer lesions. Radionuclide-labeled small-molecule PSMA inhibitors have been shown to be promising PSMA-specific agents for the diagnosis and therapy of prostate cancer. In this study, a glutamate-urea-based PSMA-targeted ligand containing an isonitrile (CNGU) was synthesized and labeled with ^99m^Tc to prepare [^99m^Tc]Tc-CNGU with a high radiochemical purity (RCP). The CNGU ligand showed a high affinity toward PSMA (*K*_i_ value is 8.79 nM) in LNCaP cells. The [^99m^Tc]Tc-CNGU exhibited a good stability in vitro and hydrophilicity (log *P* = −1.97 ± 0.03). In biodistribution studies, BALB/c nude mice bearing LNCaP xenografts showed that the complex had a high tumor uptake with 4.86 ± 1.19% ID/g, which decreased to 1.74 ± 0.90% ID/g after a pre-injection of the selective PSMA inhibitor ZJ-43, suggesting that it was a PSMA-specific agent. Micro-SPECT imaging demonstrated that the [^99m^Tc]Tc-CNGU had a tumor uptake and that the uptake was reduced in the image after blocking with ZJ-43, further confirming its PSMA specificity. All of the results in this work indicated that [^99m^Tc]Tc-CNGU is a promising PSMA-specific tracer for the imaging of prostate cancer.

## 1. Introduction

Prostate cancer is the most common cancer, and one of the leading life-threatening malignancies among men [1]. Both new cases and deaths as a result of prostate cancer have been on the rise in recent years [2]. Most prostate cancer patients with a 15% 5-year survival rate died from tumor progression in the late stage [3]. Hence, the early diagnosis of prostate cancer and detection of recurrent lesions have become one of the focuses of clinical attention all over the world [4]. Prostate-specific membrane antigen (PSMA) is glutamate carboxypeptidase II (GCPII) that is overexpressed on the surface of prostate cancer cells and plays a vital role in prostate carcinogenesis and the malignancy of the disease [5,6]. Therefore, PSMA is considered an efficient target for the nuclear imaging of diagnostics and therapeutic applications for metastatic castration-resistant prostate cancer [7,8]. Radiolabeled antibodies usually exhibit some shortcomings, such as long circulation times and a slow clearance from non-targeted organs and tissues [9]. Small molecular prostate cancer imaging agents present superior pharmacokinetics, as they accumulate quickly on tumor lesions and have a higher permeability via internalization [10]. New radioactive imaging agents bearing a glutamate-urea-based PSMA-targeted ligand are considered reliable radiolabeled tracers in clinical trials [11]. Hence, inhibitors of PSMA with a similar structure have attracted a great amount of attention for prostate cancer diagnosis and radioligand therapy, and are conjugated with various radionuclides (^99m^Tc, ^64^Cu, ^177^Lu, ^45^Ti, ^111^In, ^18^F, ^68^Ga) [12,13,14,15,16,17,18,19,20,21]. Among them, technetium-99m (^99m^Tc) is a common radionuclide for single photon emission computed tomography (SPECT), owing to its excellent nuclear physical characteristics (Eγ = 140 keV, T_1/2_ = 6.02 h). Moreover, ^99m^Tc can be obtained from a ^99^Mo/^99m^Tc generator easily, and the ^99m^Tc-labeled tracers can be prepared through kit formulation for clinical application. Though several ^99m^Tc-labeled PSMA inhibitors have been developed and evaluated in preclinical studies for prostate cancer detection [22,23,24,25,26,27], these studies have reported that ^99m^Tc-labeled PSMA inhibitors have some limitations, such as high kidney uptake, requiring further purification after radiolabeling, and having unclear structures. Thus, it is necessary to develop easily available ^99m^Tc-labeled PSMA inhibitor for the routine clinical imaging of prostate cancer.

Isonitrile (CN-R), as a bifunctional monodentate ligand, can coordinate with a ^99m^Tc(I) core to form stable octahedral complexes [28,29,30,31]. Due to the d^6^ low spin configuration of ^99m^Tc(I) in ^99m^Tc-labeled isocyanide complexes, six coordinated complexes with a great stability and inertness, such as [^99m^Tc(CN-R)_6_]^+^, can be successfully obtained. We were encouraged to prepare a stable ^99m^Tc-labeled, urea-based, PSMA-targeted ligand containing an isonitrile group and investigate the PSMA-specific imaging of prostate cancer in a mouse model. In this study, a novel urea-based PSMA inhibitor containing an isonitrile (CNGU) was synthesized and labeled with ^99m^Tc to prepare [^99m^Tc]Tc-CNGU. The potential of [^99m^Tc]Tc-CNGU for the imaging of prostate cancer was also evaluated.

## 2. Results

### 2.1. *Chemistry and Radiolabeling*

**Scheme 1:**
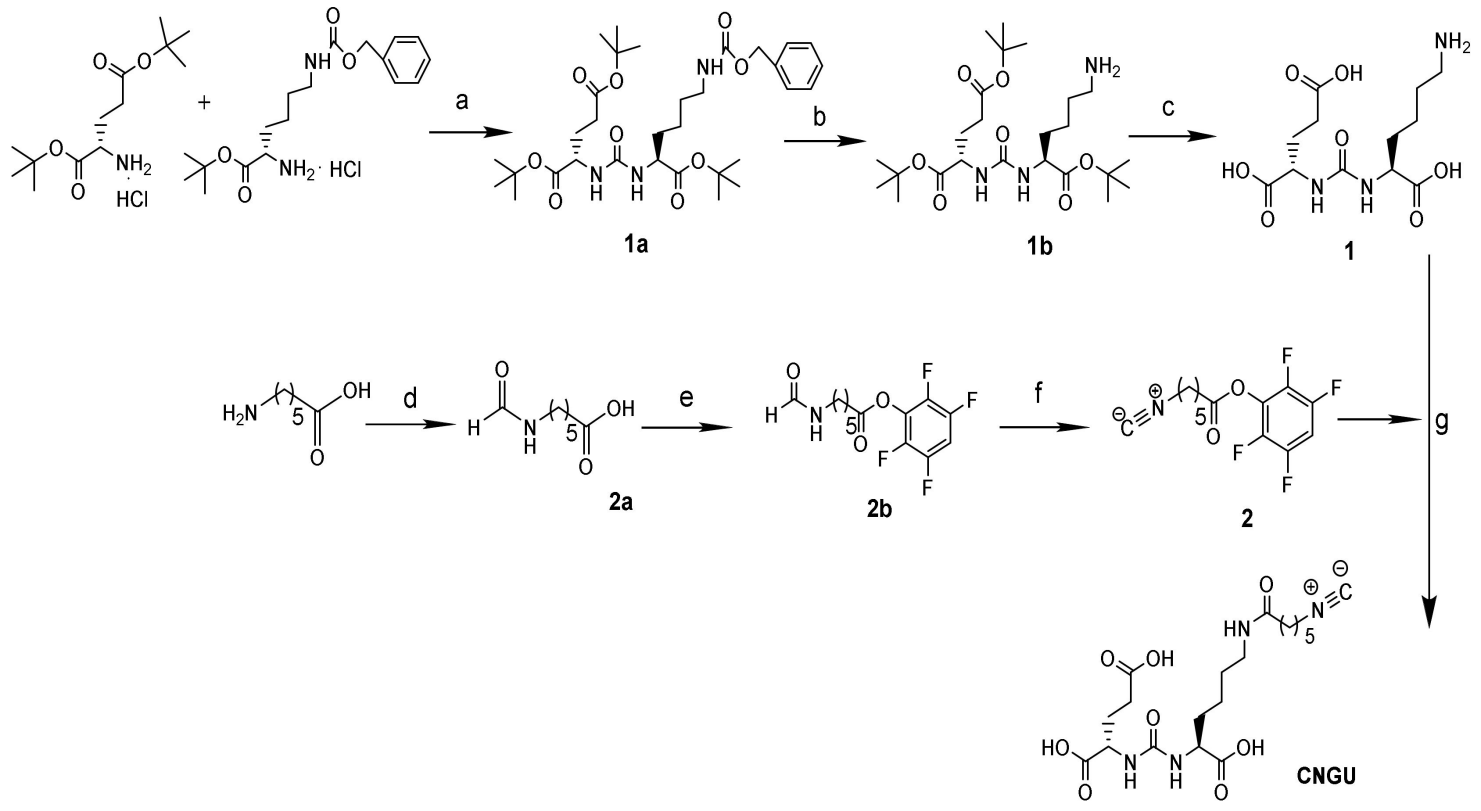
Synthetic route of the CNGU ligand. Reagents and conditions: (**a**) Triphosgene, TEA, CH_2_Cl_2_, −80 °C to r.t.,10 h; (**b**) H_2_, Pd/C, MeOH, r.t., 16 h; (**c**) TFA: CH_2_Cl_2_= 1:1; (**d**) Formic acid, DMF, 110 °C, 7 h; (**e**) 2,3,5,6-tetrafluorophenol, DCC, DMF, r.t., 8 h; (**f**) Burgess reagent, CH_2_Cl_2_, r.t., 7 h; (**g**) TEA, CH_3_OH, r.t., 24 h.

The synthesis route is shown in Scheme 1. Compound **1** and Compound **2** were prepared according to previous studies, with a slight modification [28,32].

**Figure 1:**
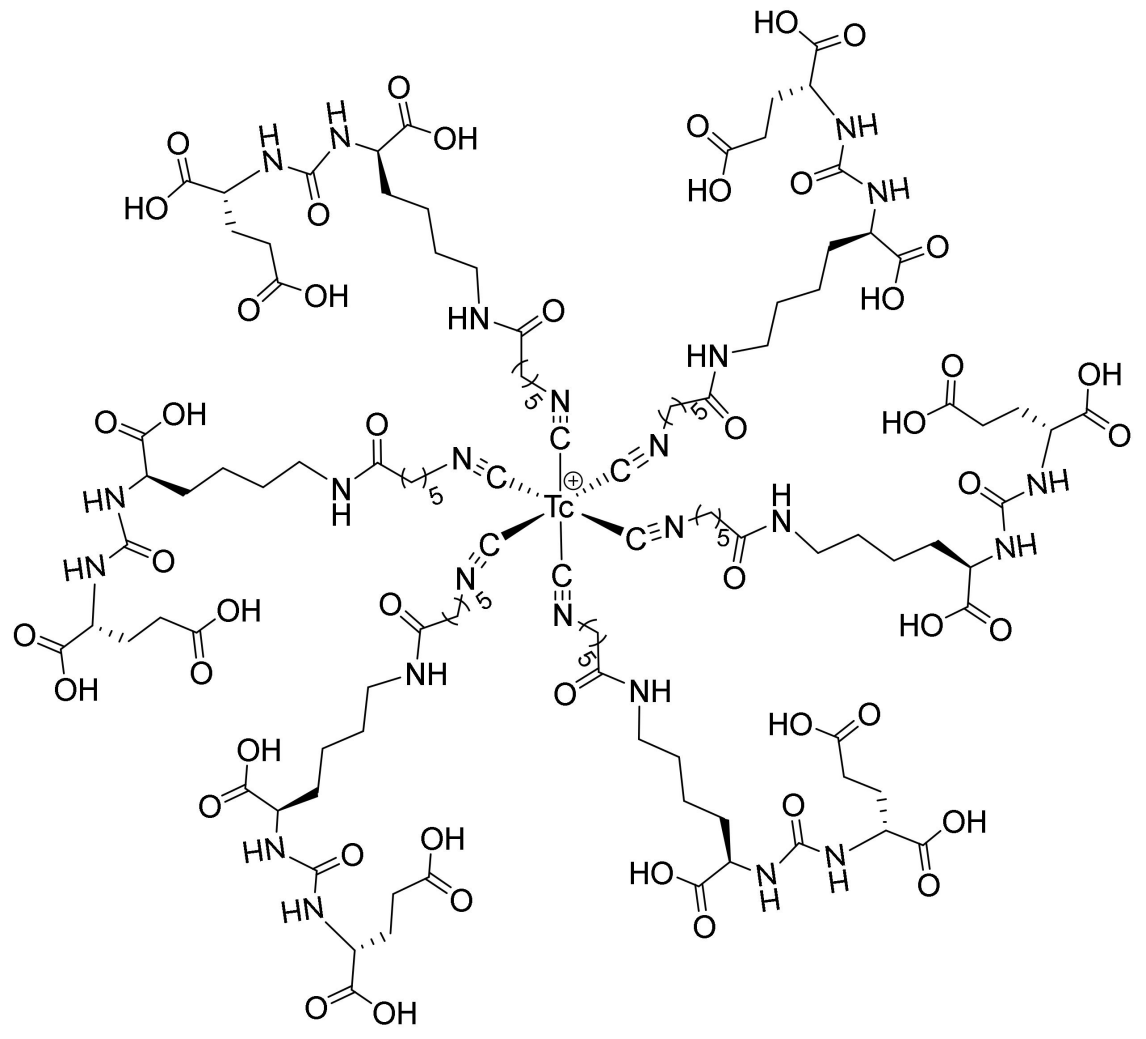
The proposed structure of [^99m^Tc]Tc-CNGU.

The final product (CNGU) was identified by Nuclear Magnetic Resonance Spectra (^1^H-NMR, ^13^C-NMR), High Resolution Mass Spectrum (HRMS), and Infrared Radiation (IR) Spectrum. The CNGU ligand was radiolabeled with ^99m^Tc in a simple way and [^99m^Tc]Tc-CNGU was easily obtained without further purification. The radiochemical purity (RCP) of [^99m^Tc]Tc-CNGU was assessed by thin layer chromatography (TLC) with a high RCP ( 97.3 ± 0.06%). The specific activity was approximately 8.22 × 10^5^ GBq/mol. The proposed structure of [^99m^Tc]Tc-CNGU is shown in Figure 1.

### 2.2. Stability and Partition Coefficients

The stability of [^99m^Tc]Tc-CNGU was assessed in saline at room temperature and in mouse serum at 37 °C. The TLC results revealed that the RCP of the complex remained at more than 90% after a 6 h incubation either in the reaction mixture at room temperature or in mouse serum at 37 °C, indicating the good in vitro stability of [^99m^Tc]Tc-CNGU (RCP ≥ 93.5 ± 0.09%). According to the determination results of the partition coefficient between octanol and PBS (pH 7.4), *P* = cpm in octanol/cpm in PBS, [^99m^Tc]Tc-CNGU (log *P* = −1.97 ± 0.03) was hydrophilic, which is due to the nature of the glutamate-urea based PSMA-targeted motif. The log *P* values of some known glutamate-urea-based PSMA-targeted inhibitors are shown in Table 1.

### 2.3. In Vitro Cell Experiments

**Figure 2:**
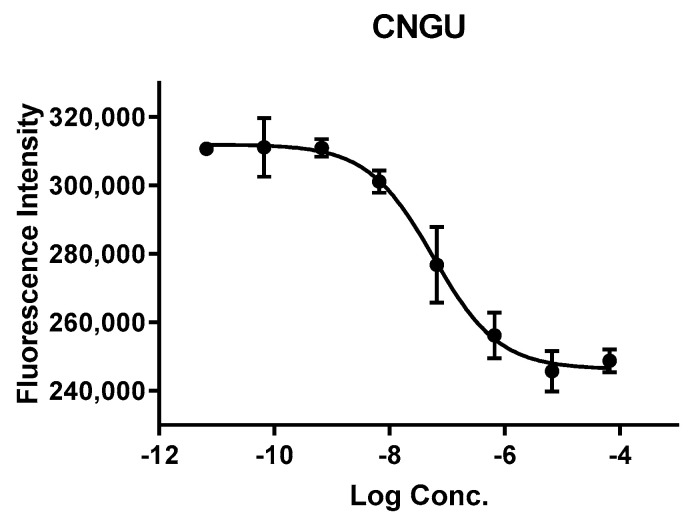
IC_50_ curve of CNGU from a fluorescence-based NAALADase assay.

An NAALADase assay was conducted to determine the binding affinity of a CNGU ligand using LNCaP cell lysates. The inhibition result (Figure 2) showed that the CNGU had a high binding affinity to the PSMA, with an enzyme inhibitory constant (*K*_i_ value) of 8.79 nM. The *K*_i_ of [^99m^Tc]Tc-CNGU may be different to that of the ligand molecule. This needs further investigation.

### 2.4. Biodistribution Study

The result of the biodistribution study of [^99m^Tc]Tc-CNGU in LNCaP (PSMA^+^) tumor-bearing mice is shown in Table 2. As anticipated, the tumor uptake of [^99m^Tc]Tc-CNGU was 4.86 ± 1.19% ID/g at 1 h post-injection, and significantly reduced to 1.74 ± 0.90% ID/g after a pre-injection of the selective PSMA inhibitor ZJ-43, suggesting that it was specific to PSMA. The kidney, as a PSMA-expressing and excretory organ, exhibited the highest uptake at 1 h post-injection. In the blocking study, the kidney and spleen uptakes could also be blocked. The liver and intestine uptakes were low, demonstrating that it was excreted through the renal system rather than the hepatobiliary route. The low uptake in the thyroid and stomach for [^99m^Tc]Tc-CNGU suggested that it was stable in vivo.

### 2.5. SPECT Imaging

**Figure 3:**
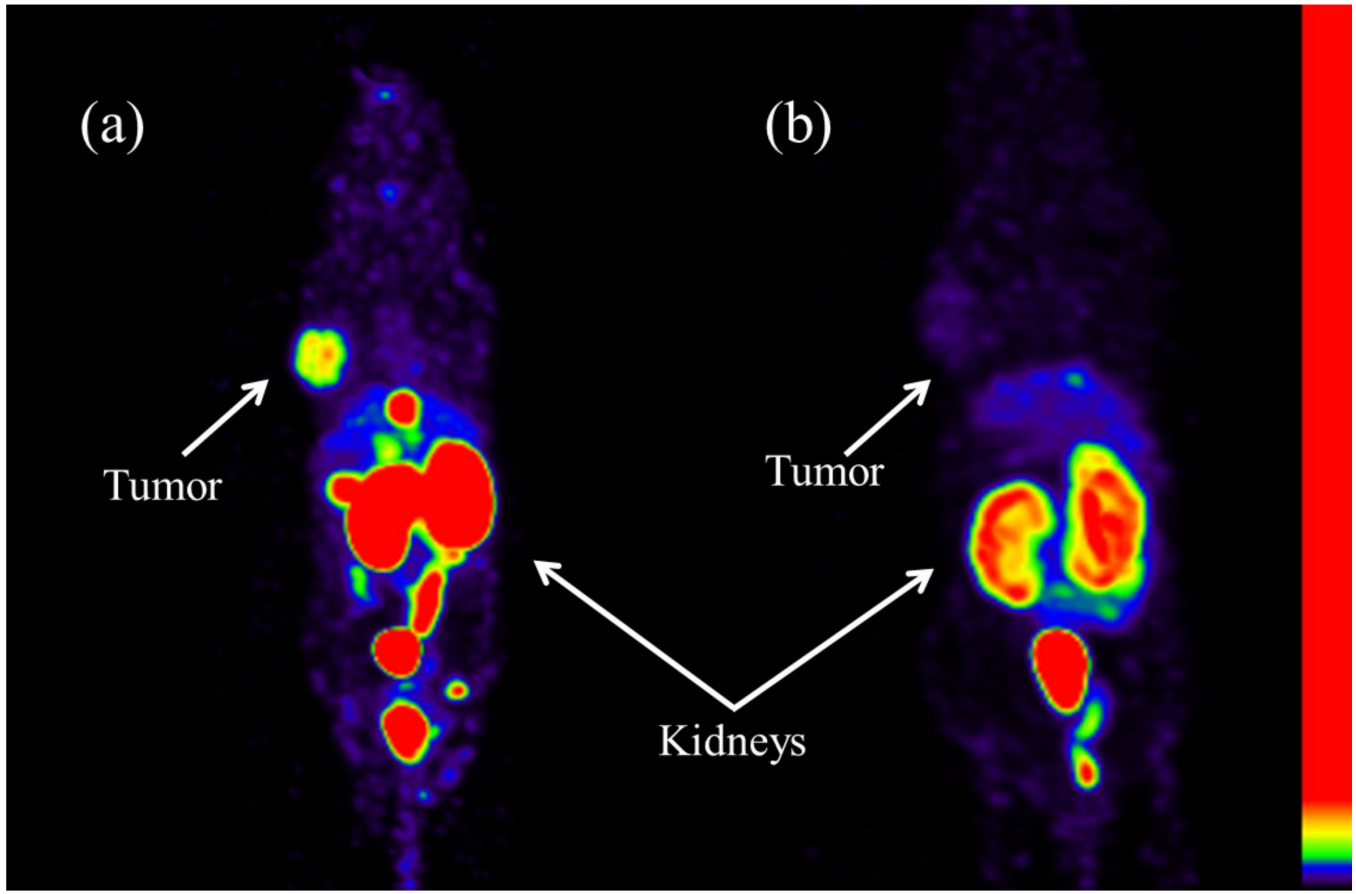
SPECT imaging of [^99m^Tc]Tc-CNGU in PSMA^+^ LNCaP tumor-bearing mice 1 h post-injection. (**a**) [^99m^Tc]Tc-CNGU. (**b**) Blockade by PSMA inhibitor ZJ-43 (500 µg).

In order to further evaluate the property of [^99m^Tc]Tc-CNGU to target PSMA in vivo, SPECT imaging in LNCaP tumor-bearing BALB/c male nude mice was carried out (Figure 3). The SPECT/CT images were consistent with the biodistribution results. The tumor and kidneys could be clearly seen from the SPECT images of [^99m^Tc]Tc-CNGU 1 h post-injection. Besides this, the uptake of the tumor and kidneys could be significantly blocked by the PSMA inhibitor ZJ-43, further confirming the PSMA specificity.

## 3. Discussion

In the synthesis process of a glutamate-urea based PSMA-targeted ligand containing an isonitrile (CNGU), the following details should be discussed. Both Compound **1** and CNGU should be purified by high performance liquid chromatography (HPLC) with the UV–Vis detector (220 nm). Because the CNGU was sensitive to temperature and acid, it should be synthesized at room temperature and purified without trifluoroacetic acid. Finally, it was able to be lyophilized to yield a pure product.

For SPECT imaging, ^99m^Tc is a perfect choice because of its ideal nuclide properties, availability, and affordability. Moreover, the ^186^Re/^188^Re analogs of the ^99m^Tc-labeled tracer can provide therapeutic tactics [33]. Thus, developing novel ^99m^Tc-labeled PSMA inhibitors for the routine clinical imaging of prostate cancer still has great significance.

A number of studies of ^99m^Tc labeled glutamate-urea-based PSMA-targeted inhibitors have been reported in recent years. In 2013, ^99m^Tc-MIP-1404 was an investigational radiopharmaceutical with good biochemical properties. It was evaluated in vivo and in clinical studies, such as in whole-body PSMA tumor detection and the biochemical recurrence of prostate cancer. However, the preparation of ^99m^Tc-MIP-1404 needs a post-labeling purification, thus restricting its wide clinical application [26,27,32]. After four years, two ligands were synthesized containing a hydrazinonicotinamide (HYNIC) chelator for the complexation of ^99m^Tc [22,23,24,25]. ^99m^Tc-EDDA/HYNIC-iPSMA and ^99m^Tc-HYNIC-ALUG can be prepared through a kit formulation without additional purification procedures. However, from the chemical point of view it was difficult to ascertain the structures of these ^99m^Tc-HYNIC complexes, which impeded the approval of the new drug. In 2019, ^99m^Tc-PSMA-11 was successfully prepared, but the radiochemical yield of the complex was lower than 90% and the purification step was essentially performed to remove ^99m^Tc-colloid [13]. In 2020, ^99m^Tc-tricabonyl-labeled glutamate-urea-based conjugates containing isonitrile ([^99m^Tc]Tc-**15** and [^99m^Tc]Tc-**16**) for the imaging of prostate cancer were prepared [30]. [^99m^Tc]Tc-**16** exhibits potential to be a promising PSMA-specific tracer. However, the preparation of [^99m^Tc]Tc-**16** needs purification by HPLC, resulting in its limited clinical application. In the present study, isonitrile (CN-R), as a strong coordination ligand, is able to chelate to ^99m^Tc to form a stable [^99m^Tc(CN-R)_6_]^+^ complex in a high yield. The preparation of [^99m^Tc]Tc-CNGU is easy, convenient, and can guarantee a straight translation into a freeze-dried kit formulation for clinical application. The RCP of the product was (97.3 ± 0.06%). There was nearly no ^99m^TcO_4_^−^ or ^99m^TcO_2_·nH_2_O and a very small amount of CNGU ligand (20 μg) in the product solution, so that further purification is not necessary before clinical usage. The structure of [^99m^Tc]Tc-CNGU would be similar to that of ^99m^Tc-CN5DG, with six CNGU ligands around the technetium-99m (+ 1) to form an octahedron structure [29].

Although many ^99m^Tc-labeled glutamate-urea-based PSMA-targeted inhibitors have been evaluated as prostate cancer imaging agents, direct comparison is difficult due to the different experimental conditions. A comparison between [^99m^Tc]Tc-CNGU and other ^99m^Tc-labeled glutamate-urea-based PSMA-targeted inhibitors is shown in Table 1. It should be noted that this is a relative comparison due to the difference in tumor and animal models. In this study, from the results of an ex vivo biodistribution study the [^99m^Tc]Tc-CNGU exhibited tracer distribution and physiological uptake on a tumor 1 h post-injection, and the accumulation of [^99m^Tc]Tc-CNGU could be blocked by PSMA inhibitor ZJ-43, indicating that the uptake was PSMA-specific. Moreover, the kidney and spleen uptakes were also blocked by a pre-injection of ZJ-43. Similar results have been recently reported. Lodhi, N. A. et al. reported a similar PSMA-targeted inhibitor, [^99m^Tc]Tc-**16**. The kidney and spleen uptakes 1 h post-injection were blocked (24.66 ± 2.17% ID/g vs. 5.66 ± 0.63% ID/g and 3.40 ± 0.93% ID/g vs. 0.40 ± 0.03% ID/g, respectively) [30]. With the low uptakes of the liver and intestine, [^99m^Tc]Tc-CNGU could improve the accuracy of prostate cancer imaging. The highest kidney uptake and the low liver uptake indicated that the main clearance route was through the urinary tract. The reason for the high kidney uptake of [^99m^Tc]Tc-CNGU is considered to be associated with the hydrophilicity of [^99m^Tc]Tc-CNGU and the overexpression of PSMA in the kidneys. As a result of this study, a reliable freeze-dried kit formulation is now available for the routine preparation of [^99m^Tc]Tc-CNGU, warranting further clinical study.

## 4. Materials and Methods

### 4.1. General Methods

All the other solvents and chemical reagents were purchased from commercial sources and used without further purification. The ^99^Mo/^99m^Tc generator was obtained from Atomic High Tech Co. Ltd. (Beijing, China). Thin layer chromatography (TLC) was performed on a polyamide strip (Luqiao Sijia Biochemical Plastic Factory, Taizhou city, Zhejiang Province, China) and eluted with ammonium acetate (1 mol/L)/methanol = 2:1 (*v*/*v*). The NMR spectra were recorded on a 400 MHz JNM-ECS spectrophotometer (JEOL, Tokyo, Japan). The mass spectra (MS) were recorded on an AB SCIEX TripleTOF™ 5600 spectrometer (AB Sciex, Concord, Canada). The SPECT studies were carried out on micro-SPECT/CT equipment (Trifoil, California, CA, USA). The radioactivity was recorded on a WIZARD2 2480 Automatic Gamma Counter (PerkinElmer, Massachusetts, MA, USA). HPLC purification was performed using a Venusil MP C18 10 µm (21.2 mm × 250 mm) preparative column (Bonna-Agela Technologies, Tianjin, China). FL-H050G preparative chromatography system (Bonna-Agela Technologies, Tianjin, China). The product was eluted using solvent A (water) and solvent B (acetonitrile). The collected HPLC eluate containing the desired ligand was lyophilized using a Pilot1-2LD freeze drier (Youshi-bio, Beijing, China). BALB/c nude mice were purchased from Peking University Health Science Center (Beijing, China). All the biodistribution experiments and SPECT imaging studies were performed in accordance with the national laws and in conformity with the guidelines of the Ethics Committee of Beijing Normal University.

### 4.2. Synthesis

The synthesis of CNGU is depicted in Scheme 1. Compound **1** and Compound **2** were synthesized according to the reported literature [28,32]. The procedures are briefly described as follows.

Compound **1**: L-glutamic acid di-tertbutyl ester hydrochloride, triethylamine, and triphosgene were mixed at −80 °C. H-Lys(Z)-Ot-Bu hydrochloride and triethylamine were added to the mixture. The crude product was purified by silica gel column chromatography to yield compound **1a.** Compound **1b** was obtained when Compound **1a** reacted with hydrogen. Compound **1b** was dissolved in a mixture of TFA: CH_2_Cl_2_ = 1:1. After reduced pressure rotary evaporation, the crude product was purified by HPLC to yield Compound **1**.

Compound **2**: 6-Aminohexanoic acid and formic acid were dissolved into DMF to yield Compound **2a**. 2,3,5,6-tetrafluorophenol was mixed with Compound **2a,** followed by DCC. Compound **2b** was obtained and reacted with Burgess reagent to yield Compound **2** after purification with silica gel column chromatography.

Compound **1** (191 mg, 0.6 mmol) was added to 25 mL of distilled water, followed by the addition of TEA (243 mg, 2.4 mmol), and stirred evenly. Compound **2** (557 mg, 1.93 mmol) was dissolved in 35 mL of methanol, which was added dropwise to said solution over a period of 1 h. The mixture was stirred for 24 h at room temperature. Most of the organic solvent was evaporated under reduced pressure, and the residue was further purified by HPLC (the mobile phases consist of water (solvent A) and acetonitrile (solvent B), with the gradient method: 0–10 min, 0% B; 10–25 min, 0–5% B; 25–35 min, 5–90% B at a flow rate of 10 mL/min) and lyophilized to obtain a brown solid (96 mg, 36.2%).

^1^H-NMR(400 MHz, DMSO-*d*_6_) δ (ppm):7.74 (t, *J* = 5.6 Hz, 1H), 6.36 (d, *J* = 8.0 Hz, 1H), 6.24 (d, *J* = 7.3 Hz, 1H), 3.97 (dt, *J* = 8.2, 4.3 Hz, 2H), 3.53–3.41 (m, 2H), 3.04 (q, *J* = 6.7 Hz, 2H), 2.98 (t, *J* = 6.3 Hz, 2H), 2.27–2.13 (m, 2H), 2.03 (dt, *J* = 9.4, 7.4 Hz, 2H), 1.74 (dd, *J* = 13.8, 6.7 Hz, 1H), 1.60 (dt, *J* = 12.9, 5.7 Hz, 1H), 1.47 (q, *J* = 7.4 Hz, 3H), 1.36 (dt, *J* = 13.3, 7.8 Hz, 3H), 1.31–1.17 (m, 4H); ^13^C-NMR(100 MHz, DMSO-*d*_6_) δ (ppm):175.08, 174.63, 174.46, 171.85, 160.94, 157.26, 52.78, 52.58, 38.38, 36.96, 35.34, 32.16, 31.44, 28.95, 28.81, 26.08, 25.01, 22.72; IR(KBr)/cm^−1^:3284 (OH/NH), 2937 (C-H), 2148 (N≡C), 1691 (C=O), 1552 (N-H), 1446 (C-N); HR-MS (ESI) for C_19_H_31_N_4_O_8_ [M + H]^+^: found 443.2140, calcd 443.2136.

### 4.3. Radiolabeling

In a vial, 100 μL of sodium citrate (10 mg/mL) and 25 μL of SnCl_2_·2H_2_O (1 mg/mL) were mixed and the pH of solution was adjusted to 6 by NaOH (0.1 M). Then, 20 μg of CNGU dissolved in 100 μL of saline was added to the solution. Afterwards, 100 μL of freshly eluted ^99m^TcO_4_^-^ (37 MBq) was added to the vial. The reaction mixture was heated at 100 °C for 20 min and cooled to room temperature. The radiochemical purity of [^99m^Tc]Tc-CNGU was determined by TLC. The TLC was carried out on a polyamide strip and eluted with ammonium acetate (1 mol/L)/methanol = 2:1 (*v*/*v*). The TLC trip was cut into 10 equal parts and the radiation counts were determined by a γ-counter. The R*f* value of ^99m^TcO_4_^−^ and ^99m^TcO_2_ nH_2_O was 0–0.1, while that of [^99m^Tc]Tc-CNGU was 0.7–1.0. All the experiments were performed in quintuplicate, and the RCP value was expressed as the mean ± standard deviation (SD).

### 4.4. Stability Studies

To determine the in vitro stability, the [^99m^Tc]Tc-CNGU was kept in labeling solution at room temperature for 6 h and then the RCP of the complex was measured by TLC. To evaluate the serum stability of the complex, the [^99m^Tc]Tc-CNGU (100 µL) was incubated in mouse serum at 37 °C for 6 h. After incubation for 6 h, 200 µL of acetonitrile was added to the sample and the mixture was centrifuged to precipitate the proteins. Afterwards, the supernatant was analyzed by TLC.

### 4.5. Determination of the Partition Coefficient

The lipophilicity of the radio-conjugate was evaluated by a routine method. Briefly, 100 μL of the complex was added to a mixture of 1-octanol (800 μL) and 0.025M PBS pH = 7.4 (700 μL). The mixture was vortexed for 2 min and centrifuged at 2000 rpm for 3 min to allow phase separation at room temperature. Aliquots of 100 μL of both 1-octanol and PBS were collected and determined by γ-counter. The partition coefficient was calculated by dividing the counts in the octanol phase by those in the PBS, and the results were expressed as log *P* = log (cpm in octanol/cpm in PBS). All the experiments were performed in triplicate, and the log *P* value was expressed as the mean ± SD (standard deviation).

### 4.6. NAALADase Assay

The PSMA inhibitory activity was analyzed according to the previously described method of fluorescence-based Amplex Red glutamic acid assay [10]. The lysates of the LNCaP cell extracts (30 μL), CNGU (15 μL, changeable concentrations from 0.01 nM to 1 μM) and *N*-acetylaspartylglutamate (3.5 μM, 15 μL) were mixed in a 96-well plate and incubated at 37 °C for 2 h. The glutamate concentration was obtained according to the result of incubating with a standard solution (50 μL) of the Amplex Red glutamic acid test kit for 30 min. The fluorescence was determined by a plate reader (λ_ex_ = 530 nm and λ_em_ = 590 nm). The inhibition curve was shown in the form of a semilog plot. The half-maximal inhibitory concentration (IC_50_ value) was determined when the PSMA enzyme activity was inhibited to 50% of the original concentration. The enzyme inhibitory constant (*K*_i_ value) was calculated with the Cheng–Prusoff equation. The NAALADase assay was performed in duplicates, and the data analysis was processed by non-linear regression using GraphPad Prism version 5.00 (GraphPad Software, San Diego, CA, USA).

### 4.7. Biodistribution Experiments

The ex vivo biodistribution of the complex was evaluated in groups of 5 BALB/c nude male mice (four-week-old) bearing LNCaP xenografts with a tumor size 6–8 mm in diameter. Mice were injected [^99m^Tc]Tc-CNGU (74 kBq, 100μL) via a tail vein and sacrificed by neck dislocation 60 min after injection. Blood samples and other samples of the main organs were collected, weighed and counted by a γ-counter. For the blocking study, the mice were pretreated with PSMA-targeted inhibitor ZJ-43 (500 µg) for 30 min and then [^99m^Tc]Tc-CNGU (74 kBq, 100μL) was injected intravenously. The mice were sacrificed 60 min post-injection and the radioactivity of their tissues was measured. The results were expressed as the percentage of the uptake of injected dose per gram of tissue (% ID/g) and the values were described as the mean ± standard deviation (SD).

### 4.8. SPECT Imaging

The SPECT images were taken in the BALB/c nude male mice bearing LNCaP xenografts (*n* = 3) with a tumor size of 6–8 mm in diameter 1 h after the injection of [^99m^Tc]Tc-CNGU (45 MBq, 100 μL). A blocking study was also performed in the micro-SPECT imaging study. For the blocking experiment, mice were injected with ZJ-43 (500 µg) 30 min before an injection of [^99m^Tc]Tc-CNGU (45 MBq, 100 μL). Whole-body images of the mice were obtained by SPECT, with HiSPECT, and were reconstructed by the vivoquant 2.5 software. During the scintigraphy, the mice were anesthetized with an inhalation of 1.5% isoflurane.

## Figures and Tables

**Table 1:** Comparison of the biodistribution of [^99m^Tc]Tc-CNGU and other ^99m^Tc-labeled glutamate-urea-based PSMA-targeted inhibitors.

Complex	**[^99m^Tc]Tc**- CNGU	^99m^Tc-MIP-1404	^99m^Tc-EDDA/HYNIC- iPSMA	^99m^Tc-HYNIC- ALUG	**[^99m^Tc]Tc**-16
Cell	LNCaP	LNCaP	LNCaP	LNCaP	22Rv1
Animal	BALB/c	NCr nude	Athymic mice	SCID	BALB/c
Tumor	4.86 ± 1.19	10.3 ± 2.5	10.22 ± 2.96	14.13 ± 2.95	1.87 ± 0.11
Kidneys	70.95 ± 12.28	105 ± 37	23.63 ± 3.56	197.50 ± 7.1	24.66 ± 2.17
T/B	2.89	79	62.33	11.78	4.43 ± 0.39
T/M	12.46	57	68.13	19.45	14.05 ± 1.78
log *P*	−1.97 ± 0.03	--	--	−2.68 ± 0.12	−2.10 ± 0.03
Reference	This study	[26]	[24]	[22]	[30]

T/B: Tumor/Blood; T/M: Tumor/Muscle. Radioactive accumulation in each organ or tissue is expressed as % ID/g. --: The data was not mentioned in the original literatures.

**Table 2:** Biodistribution of [^99m^Tc]Tc-CNGU in BALB/c male nude mice bearing LNCaP (PSMA^+^) tumor 1 h post-injection.

**Organs**	**1 h**	**1 h Blockade**
Heart	0.74 ± 0.15	0.46 ± 0.04
Liver	2.46 ± 0.72	1.48 ± 0.16
Spleen	5.84 ± 1.51	0.78 ± 0.19 *
Lung	1.89 ± 0.30	1.47 ± 0.19
Kidneys	70.95 ± 12.28	19.85 ± 1.30 *
Stomach	0.64 ± 0.21	0.44 ± 0.14
Bone	0.43 ± 0.11	0.40 ± 0.08
Intestine	2.16 ± 0.34	2.19 ± 0.37
Pancreas	0.86 ± 0.16	0.34 ± 0.04
Muscle	0.39 ± 0.06	0.24 ± 0.01
Tumor	4.86 ±1.19	1.74 ± 0.90 *
Blood	1.68 ± 0.33	1.30 ± 0.10
Thyroid (% ID)	0.01 ± 0.00	0.01 ± 0.00
Tumor/Blood	2.89	1.34
Tumor/Muscle	12.46	7.25

Radioactive accumulation in each organ or tissue (thyroid was evaluated as % ID) is expressed as % ID/g (mean ± SD for *n* = 5). The blocking studies were performed by a pre-injection of ZJ-43 (500 µg). * *p* < 0.05.
